# The Role of Lymphocytes in Radiotherapy-Induced Adverse Late Effects in the Lung

**DOI:** 10.3389/fimmu.2016.00591

**Published:** 2016-12-14

**Authors:** Florian Wirsdörfer, Verena Jendrossek

**Affiliations:** ^1^Institute of Cell Biology (Cancer Research), University Hospital Essen, Essen, Germany

**Keywords:** lymphocytes, radiotherapy, lung, pneumonitis, fibrosis

## Abstract

Radiation-induced pneumonitis and fibrosis are dose-limiting side effects of thoracic irradiation. Thoracic irradiation triggers acute and chronic environmental lung changes that are shaped by the damage response of resident cells, by the resulting reaction of the immune system, and by repair processes. Although considerable progress has been made during the last decade in defining involved effector cells and soluble mediators, the network of pathophysiological events and the cellular cross talk linking acute tissue damage to chronic inflammation and fibrosis still require further definition. Infiltration of cells from the innate and adaptive immune systems is a common response of normal tissues to ionizing radiation. Herein, lymphocytes represent a versatile and wide-ranged group of cells of the immune system that can react under specific conditions in various ways and participate in modulating the lung environment by adopting pro-inflammatory, anti-inflammatory, or even pro- or anti-fibrotic phenotypes. The present review provides an overview on published data about the role of lymphocytes in radiation-induced lung disease and related damage-associated pulmonary diseases with a focus on T lymphocytes and B lymphocytes. We also discuss the suspected dual role of specific lymphocyte subsets during the pneumonitic phase and fibrotic phase that is shaped by the environmental conditions as well as the interaction and the intercellular cross talk between cells from the innate and adaptive immune systems and (damaged) resident epithelial cells and stromal cells (e.g., endothelial cells, mesenchymal stem cells, and fibroblasts). Finally, we highlight potential therapeutic targets suited to counteract pathological lymphocyte responses to prevent or treat radiation-induced lung disease.

## Introduction

About 60% of all cancer patients receive radiotherapy (RT) at some point during the course of their disease, and good results in terms of long-term survival and tumor cure are achieved in a variety of tumors by multimodal combinations of surgery, RT, and chemotherapy. Concurrent radiochemotherapy could improve the prognosis of glioma, lung, head and neck, esophageal, cervical, anal, and rectal cancer (1–8) and is part of standard therapy for locally advanced tumors of these entities. Yet, treatment outcome is still unsatisfactory for common forms of cancer with high loco-regional failure rates or frequent development of metastases. Although patient-specific clinical factors may explain some of these failures, it is commonly assumed that biological factors adversely affecting the response of tumor cells to treatment, such as intrinsic radioresistance, tumor promoting mutations, unfavorable gene expression profiles, heterogeneity in radiation responses, or a resistance-promoting microenvironment, significantly contribute to treatment failures (9–14). Acute and late toxicity to normal tissues also limits the radiation dose that can be applied to the tumor, and tolerable doses are often linked to suboptimal tumor control—even accepting side effects that lead to decreased quality of life (15). Normal tissue toxicity also precludes therapy intensification efforts for many locally advanced tumors by the combination with cytotoxic chemotherapy (16–18). As a consequence, there is high interest in improving the therapeutic ratio either by technical and physical innovations in treatment delivery, e.g., intensity-modulated radiation therapy or particle therapy, or by developing effective strategies to prevent or treat the toxic effects of ionizing radiation (IR) in normal tissues without protecting the tumor cells, or to increase intrinsic radiosensitivity of cancer cells without increasing sensitivity of normal tissue cells, respectively.

**Figure 1:**
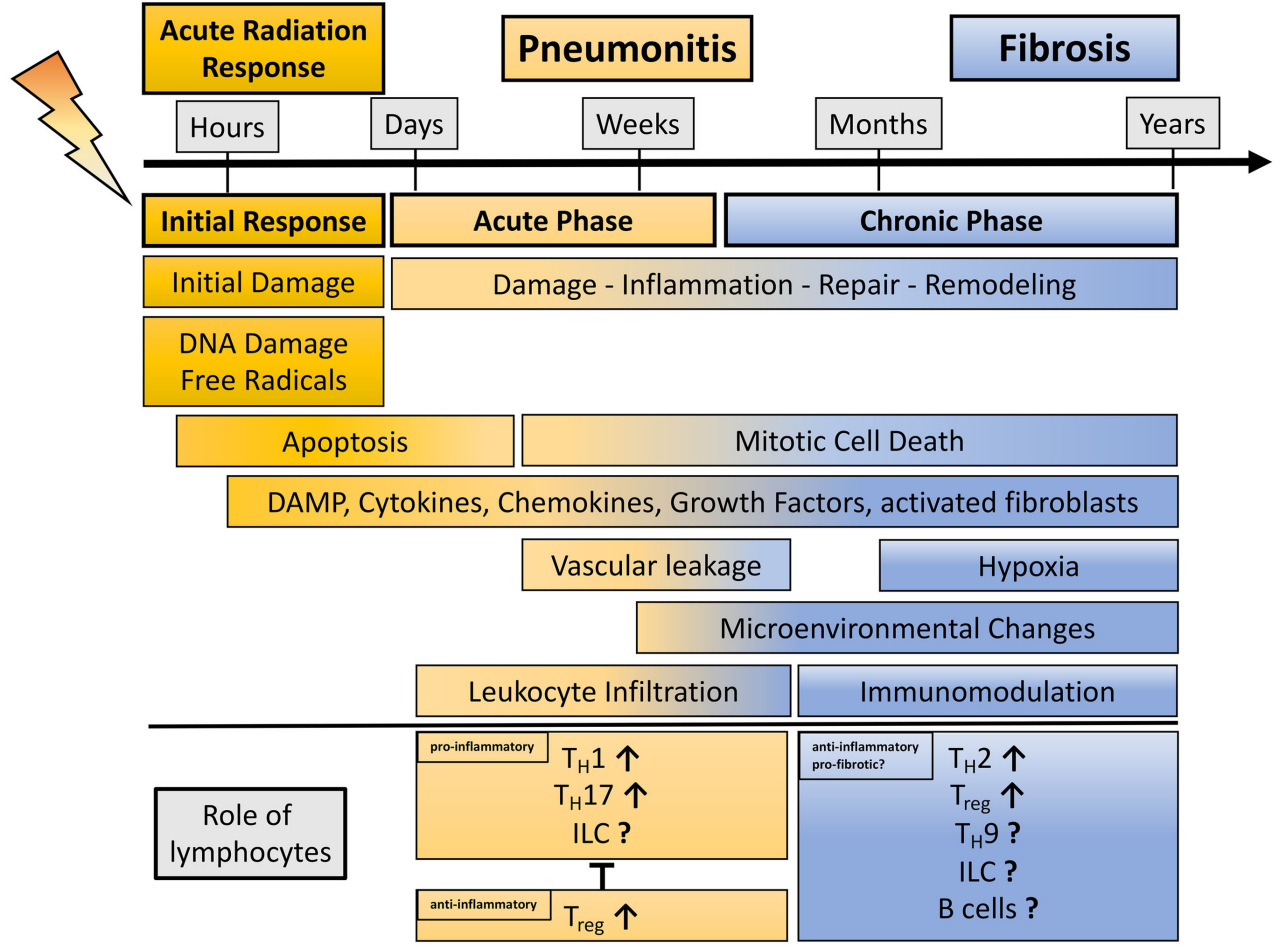
**Schematic representation showing the phases of radiation-induced lung injury over time with a view on the dual role of lymphocytes during radiation-induced pneumopathy**. Damage to the lung results in an initial response (acute radiation response) due to DNA damage, ROS induction, and apoptosis. Release of damage-associated molecular patterns (DAMPs) and secretion of cytokines and chemokines activate the immune system. This phase passes over into an acute inflammatory phase (pneumonitis) that is characterized by an enhanced pro-inflammatory response and vascular leakage. In this phase, diverse lymphocyte subpopulations like T_H_1, T_H_17, and potentially innate lymphoid cells (ILC) can contribute to inflammation, whereas it is believed that the lymphocyte subpopulations T_reg_ are needed to control harmful, excessive pro-inflammatory responses. Resolution of inflammation and repair induction is paralleled by late mitotic cell death subsequent, hypoxia, release of DAMP, cytokines, and growth factors. These alterations in the lung micromilieu are described for the chronic phase of radiation-induced pneumopathy. These environmental changes can contribute to immunomodulation; here, it is believed that lymphocytes (T_H_2, T_H_9, T_reg_, and potentially ILC) show an anti-inflammatory or even pro-fibrotic phenotype, thereby having the potential to further alter the environment in the lung toward the induction of disease-promoting myofibroblasts and fibrosis development.

Dose-limiting side effects in the lung tissue after RT of the thoracic region or total body irradiation in conditioning regimens for hematopoietic stem cell transplantation include inflammatory (pneumonitis) and fibrotic changes (pulmonary fibrosis) (19–21). Radiation-induced damage to the lung tissue leads, like infectious, thermal, or physical damage, to the activation of the immune system. This inflammatory response is needed to orchestrate tissue repair and regeneration in order to restore tissue homeostasis. Depending on the degree of the resulting aseptic inflammation, patients can present with pneumonitis. Radiation-induced pneumonitis can develop at 4–12 weeks after RT with symptoms like fever, chest pain, dry cough, and dyspnea or even respiratory failure in severe cases and occurs in 5–20% of patients with lung or breast cancer (22–24). The pneumonitic phase is characterized by the recruitment of diverse immune cells of myeloid and lymphoid origin and a perpetual cascade of cytokines/chemokines resulting in various degrees of lung inflammation and the described symptoms (Figure 1).

**Figure 2:**
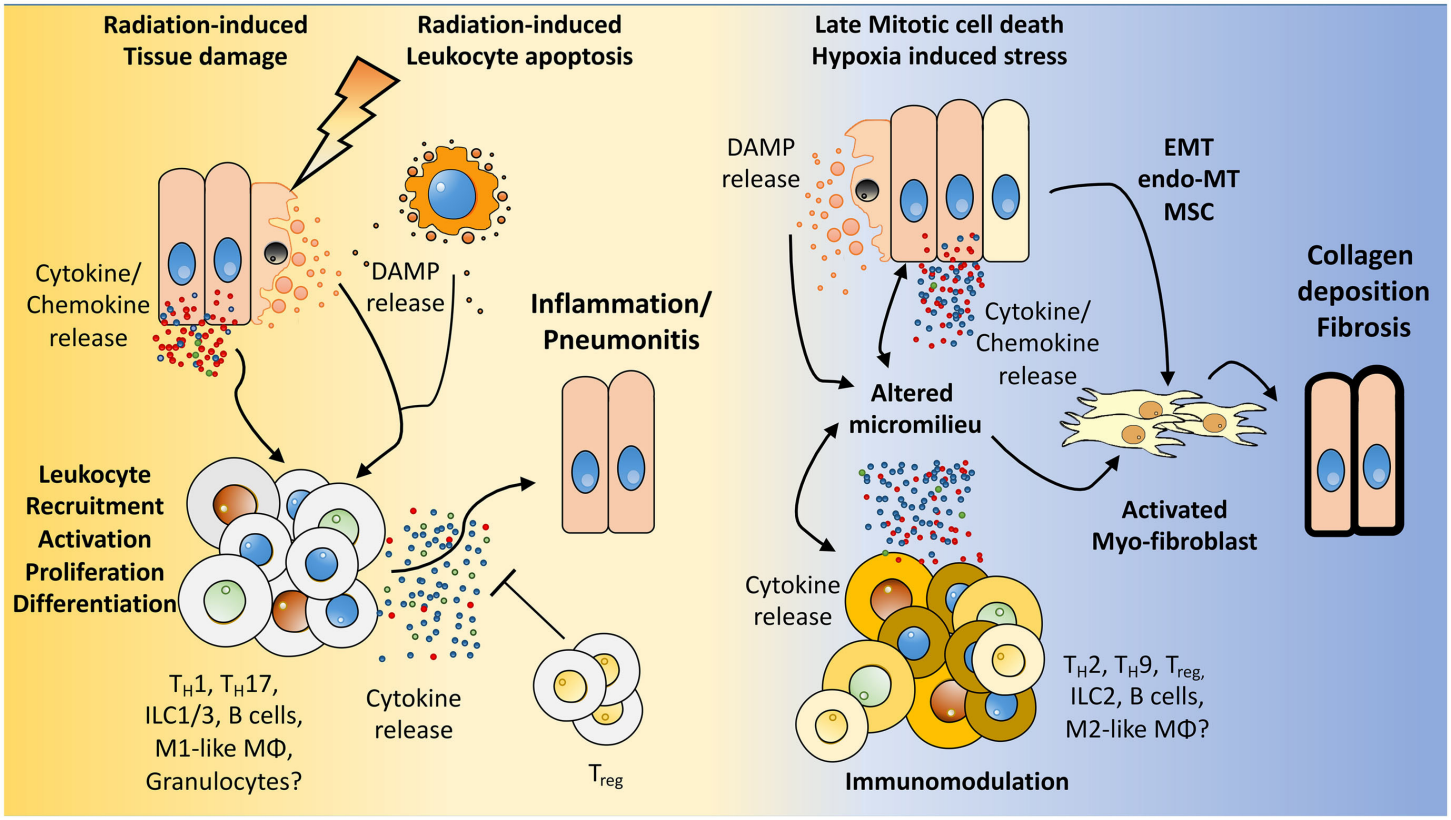
**How the microenvironment shapes the immune response and *vice versa***. We hypothesize that radiation induces damage to tissue resident cells, e.g., endothelial and epithelial lung cells, mesenchymal stem cells (MSC) as well as in resident immune cells. The resulting tissue damage can initiate stress responses or cell death with subsequent release of cytokines/chemokines and damage-associated molecular patterns (DAMPs). This initial damage response leads to the recruitment and activation of diverse immune cells to the lung, among them lymphocytes. Further activation, proliferation of these cells, and secretion of cytokines shape the pulmonary micromilieu toward inflammation and—if this response is too excessive—to the development of severe pneumonitis. Late chronic mitotic cell death and subsequent tissue hypoxia lead to the release of DAMPs and cytokines/chemokines from resident cells thereby altering the micromilieu in the lung. These environmental changes impact on the immune cells present in the lung tissue and promote an altered cytokine release of immune cells. Finally, epithelial-mesenchymal-transition, endothelial-mesenchymal-transition, mesenchymal stem cell differentiation, and the altered environment contribute to the induction of activated myofibroblasts, collagen deposition, and fibrosis.

Development of radiation-induced lung fibrosis is mostly observed 6–24 months after RT and may become chronic in patients with a large irradiated lung volume (24). Major symptoms of lung fibrosis are breathing difficulties and subsequent volume loss of the lung (25). Studies from various groups using rodent models and patient samples helped to reveal a complex response of the lung tissue toward irradiation with multiple interactions between resident lung cells including lung-resident mesenchymal stem cells (MSC), locally generated or recruited fibroblasts, and infiltrating immune cells, respectively (26–37). We speculate that a sophisticated network between damaged resident cells (epithelial cells, endothelial cells, and lung-resident MSC), recruited immune cells, and soluble mediators (cytokines, chemokines, growth factors, and proteases) and the resulting environmental changes participate in shaping the observed inflammatory and fibrotic alterations of the lung tissue (Figures 1 and 2).

Among other molecular markers there is evidence from preclinical and clinical studies that T lymphocytes infiltrate the lung to a considerable extent, particularly during the pneumonitic phase at 3–12 weeks post-irradiation, although lymphocyte infiltration was also observed at later time points during the chronic inflammatory/fibrotic phase at 16–30 weeks post-irradiation (30, 35, 38–40). Interestingly, earlier studies described a correlation between the presence of CD4^+^ T cells in the bronchoalveolar lavage of irradiated breast or lung cancer patients and the development of pneumonitis (39, 41, 42). Furthermore, depletion of CD4^+^ T cells during the pneumonitic phase attenuated the development of lung fibrosis upon thoracic irradiation in a murine model (43). These findings suggest a complex role of CD4^+^ T cells in the pathogenesis of radiation-induced lung disease. Antibody-mediated inhibition of the accumulation of CD3^+^ lymphocytes or depletion of CD4 and CD8 T lymphocytes also reduced fibrosis levels in the murine model of pulmonary fibrosis induced by the radiomimetic and DNA-damaging drug bleomycin (BLM) (44, 45). In contrast, the lack of mature T and B lymphocytes in *recombination-activating gene 2* (RAG2)-deficient mice exacerbated radiation-induced fibrosis (46). Altogether, these findings highlight that lymphocytes play a complex role in DNA damage-induced lung disease and suggest that depending on the disease stage and the environmental conditions, shaped by the tissue response to the damage, specific lymphocyte subpopulations exert either beneficial or adverse effects (Figure 1). We propose that a disturbed balance between tissue inflammation and repair processes participates in the development of radiation-induced pulmonary fibrosis as it has been described for other fibrotic diseases and that lymphocytes are involved in these processes (47). Nevertheless, it remains to be demonstrated whether lymphocytes directly contribute to radiation-induced lung disease or only modulate disease progression. Furthermore, it remains to be explored whether, besides the myeloid compartment, innate lymphoid cells (ILC) might contribute to radiation-induced fibrosis. Finally, the mechanisms driving radiation-induced lymphocyte deviation remain to be defined.

## Lymphocytes: Effector Cells of the Immune System

Lymphocytes are characterized as white blood cells that are homogeneous in appearance but that have various functions. They include T cells, B cells, and ILC among them conventional natural killer (NK) cells. T cells, ILC, and B cells are responsible for the production of cytokines and antibodies (B cells), whereas NK cells can induce direct cell-mediated killing of virus-infected cells and tumor cells. Here, we will focus on a potential role of B and T lymphocytes as well as ILC.

The different major subpopulations of T lymphocytes include CD8^+^, CD4^+^ T cells, NK T cells, and γδT cells. CD8^+^ T cells comprise cytotoxic T cells or cytolytic T cells. They control and eliminate intracellular pathogens and tumor cells and can further differentiate into CD8^+^ memory cells (48). γδT cells express a T cell receptor differing from the conventional αβT cells. The function of γδT cells is poorly understood, but current knowledge implies a role in immunoregulation in pathogen and allergen responses (49). NK T cells are a unique subpopulation of lymphocytes that are mainly involved in innate immunity and will not be further discussed in the present review.

CD4^+^ T cells comprise T_H_1 and T_H_2 subpopulations. Furthermore, advances in immunology have led to the characterization of newly appreciated CD4^+^ T cell effector populations that regulate the immune response such as interleukin (IL)-17-producing T cells (T_H_17 cells), T cells with regulatory function [regulatory T cells (T_reg_)], IL-9-secreting T_H_9 cells, IL-22-dominant T_H_22 cells, and B cell-interacting follicular helper T cells (T_FH_), thus revising established paradigms (50–58).

The secretion of interferon (IFN)-γ and the directed elimination of intracellular pathogens characterize a T_H_1 response. In contrast, T_H_2 responses are shaped by the cytokines IL-4 and IL-13, supporting the defense against parasites, and moreover contribute to the generation of antibodies (59).

T_H_17 cells preferentially produce IL-17A-F and play a role in inflammatory processes such as autoimmune diseases and the defense against extracellular pathogens. T_H_17 cells further produce the cytokines IL-21, IL-22, and IL-23, which exert strong pro-inflammatory effects (60). T_H_17 cells are induced by IL-6, IL-21, and transforming growth factor beta (TGF-β), a potent regulator of lung homeostasis as well as in pathologies (61, 62).

Another important subpopulation of T cells are T_reg_. T_reg_ show a suppressive capacity, control immune reactions, and inhibit exaggerated inflammation (60, 63). T_reg_ exist as natural occurring T_reg_ (nT_reg_) and induced T_reg_ (iT_reg_). Murine thymus-derived nT_reg_ are CD4^+^/CD25^+^ and express the transcription factor FoxP3 (murine cell marker), whereas in humans not all T_reg_ express FoxP3. Therefore, T_reg_ in humans are mainly characterized *via* the marker profile CD4^+^/CD25^hi^/CD127^low^ (64). T_reg_ show their suppressive capacity by secreting anti-inflammatory cytokines like IL-10 and TGF-β, which can induce cell cycle arrest or apoptosis in T effector cells.

Recent studies also revealed a small population of CD8^+^ T_reg_ at steady state; these CD8^+^ T_reg_ are present in the human and the murine system, and they express the marker CD25 as well as FoxP3. Churlaud et al. showed that CD8^+^ T_reg_ are highly suppressive and responsive to IL-2 (65), but further studies are needed to uncover the origin and the role of CD8^+^ T_reg_ in health and pathologies.

B lymphocytes represent the second heterogeneous group of lymphocytic cells. B cells originate from the bone marrow, mature in the spleen, and differentiate in the lymph nodes into germinal center cells after contact with antigens and T cells (66, 67). Besides their capacity for antibody secretion, they have functions in antigen presentation and secretion of diverse cytokines (68, 69).

The newly identified group of the lymphoid cells, namely ILC, plays an important junction between innate and adaptive immunity. Like other lymphoid cells, they originate from a common lymphoid progenitor in the bone marrow. But in contrast to their relatives, they lack (RAG)-dependent rearrangement of antigen receptors as well as phenotypical markers of myeloid and dendritic cells (70, 71). ILC have been characterized by their expression pattern of the master transcription factors (T-bet, GATA3, and RORγt) and specific cytokines that usually define T cell subpopulations. Based on this categorization, three different subpopulations (ILC1, ILC2, and ILC3) have been defined as follows: (i) ILC1 cells include conventional NK cells and T-bet^+^/IFN-γ-producing cells; (ii) ILC2 cells show GATA3 expression, and they secrete T_H_2 cytokines, IL-4, IL-5, IL-9, and IL-13 in response to IL-25 and IL-33; and (iii) ILC3 cells express the transcription factor RORγt, and they release the cytokines IL-17, IL-22, granulocyte–macrophage colony-stimulating factor (GM-CSF) as well as lymphotoxins. ILC act on tissue homeostasis and tissue remodeling; moreover, they participate in regulating immune responses to inflammation and infection in tissues like liver, lymph nodes, and mucosal barriers like gut and lung (72, 73). These characteristics make it highly likely that ILC also modulate the progression of radiation-induced lung inflammation and fibrosis (see Role of Lymphocytes in the Defense of the Lung Tissue).

## Role of Lymphocytes in the Defense of the Lung Tissue

In the lung, T cells are found in relatively high numbers in the mucosa, in the intraepithelial part, in the underlying lamina propria, and also in the lung parenchyma. T cells of the intraepithelial region express CD8, while CD4 is the dominant surface marker of T cells in the lamina propria. Furthermore, it has been described that CD45RO is expressed on both, CD4 and CD8 T cell subsets, indicating their role as effector and/or memory cells (74). For CD8^+^ T cells it is known that they protect the lung against influenza infection. Nevertheless, there it is believed that CD8^+^ T cells also contribute to lung injury, e.g., during influenza infection due to their cytotoxic effects and the massive production of the pro-inflammatory cytokines IFN-γ and tumor necrose factor (TNF)-α (75), respectively.

Among the CD4^+^ T cells, lung T_H_17 cells may not only play a role in neutrophil recruitment and pathogen clearance but also be highly relevant in respiratory inflammatory diseases (76). Moreover, a recent study demonstrated that T_H_17 cells also play a role in lung cancer progression: here, the authors analyzed blood samples from patients and described that T_H_17 as well as T_reg_ subsets are involved in the immunopathology of NSCLC (77). In other studies, T_reg_ were found to counteract the inflammation-induced injury to the airways associated with lung infections as well as the development of atopic diseases. T_reg_ also play a role in mediating inhalation tolerance and in controlling allergen-specific T cells from activation (74, 78). Interestingly, there is evidence from some studies that T_reg_ contribute to fibrotic diseases in the lung by promoting a pro-fibrotic microenvironment (79–81).

Also, γδ T cells show functions in the airways. For the lungs it is described that γδ T cells reside in the subepithelium of alveolar and non-alveolar regions (82). Here, they modulate immune responses against allergens and pathogens (49, 83). A study from Simonian et al. also revealed a role in inflammation (hypersensitivity pneumonitis)-induced lung fibrosis. Here, γδ T cells diminished CD4^+^ cell recruitment by the secretion of regulatory IL-22 thereby protecting the lung from fibrosis (84).

However, the role of T_H_9, T_H_22, and T_FH_ cells in lung pathologies is still underexplored. So far, current studies hint to a role for T_H_22 and T_FH_ cells in host defense against viruses and bacteria in the lung, whereas T_H_9 cells seem to play an important role in asthma (85–87).

Similar to T cell populations, B cells are also present in the parenchymal lung as well as in the conducting airways. In the lamina propria, they act as antibody-secreting plasma cells producing immunoglobulin (Ig)A but may also contribute to local antigen presentation (74, 88). In the lung, B cells can act, for example, as memory B cells producing IgA and IgG neutralizing antibodies that have the ability to protect against pulmonary viral reinfections (89).

The group of ILC is in the focus of current research. ILC have been implicated in immunity of the mucosal barrier in the lung, e.g., in allergic asthma, hyper responsiveness, and viral infection (90–93). The present knowledge on ILC has nicely been summarized in two recent reviews with a focus on their roles in the lung tissue (94, 95). Though these reviews emphasize that the role of ILC in the lung is still poorly characterized, we will highlight some important observations at this point. Among the three ILC subsets defined so far, ILC2 present the main ILC in the murine lung, but with 2–3 × 10^4^ cells per lung and thus 0.4–1% of total lung cells, these ILC seem to be a relatively rare population, at least under physiological conditions (91). But, pathological conditions in the lung are associated with changes in the ILC population (96). In this context, ILC2 and ILC3 seem to play more imported roles than ILC1 during chronic lung disease in both, mice and men (94, 97, 98). For example, in patients with chronic obstructive pulmonary disease (COPD) ILC3 constitute the major population with 60% of all ILC, whereas ILC2 amount to 30% and ILC1 to 10% of the ILC (98). Of note, depletion of ILC2 reduced epithelial integrity, induced epithelial degeneration, and impaired lung functions during influenza virus infection highlighting a protective role of ILC2 for these processes (91).

First reports suggest that ILC may also play an important role during acute pulmonary inflammation. In this context, ILC3 have recently been identified as a major source of IL-17A thereby inducing neutrophil recruitment in a murine model of LPS-induced acute respiratory distress syndrome (99). Furthermore, in eosinophilic crystalline pneumonia, a murine idiopathic type 2 lung inflammation, IL-2 was shown to function as an important activator of ILC2 functions; the authors further highlight a potential cross talk between ILC2 and ILC3 as well as T cells and T_reg_ during disease pathogenesis (100). Even more important, in a murine model for allergic asthma, pulmonary epithelial cell-derived TGF-β1 and IL-33 contributed to ILC2-mediated responses (101).

Besides their effects on acute pulmonary responses, ILC also impact the development of pulmonary fibrosis [for a recent review, refer to Ref. (102)]. In this context, an interplay between ILC, macrophages, and cells of the adaptive immune system was shown to participate—together with IL-13, IL-25, and IL-33—in the pathogenesis of BLM-induced pulmonary fibrosis in mice (103–105). Moreover, Hams et al. also found elevated levels of IL-25 and ILC2 in the lungs of patients with idiopathic pulmonary fibrosis (IPF) (105, 106), whereas others described a role for ILC2 and ILC3 in fibroblast activation providing a mechanistic link between ILC and fibrotic diseases (102).

Since work from our own laboratory and others implicate T_H_17 cells and T_reg_ in the pathogenesis of radiation-induced lung disease, these observations strongly suggest that ILC, particularly ILC2 and ILC3, might also participate in the cross talk between damaged resident cells, recruited immune cells, and activated fibroblasts and thus modulate the extent of lung inflammation and progression of radiation-induced fibrosis. But, an involvement of ILC in radiation-induced pneumopathy remains to be demonstrated, and potential beneficial or disease-promoting effects during the different disease stages (inflammation, fibrosis) have to be explored.

## Impact of Ionizing Radiation on Lymphocytes

Radiation therapy is an essential and common approach in cancer treatment. So far, the use of IR in cancer treatment is based on its high potential to induce tumor cell death and to abrogate survival of clonogenic tumor cells. The toxic effects of IR result from the deposition of energy from IR in tumor and normal tissue cells including immune cells. Energy deposition results in damage to cellular macromolecules, particularly cellular DNA, e.g., by direct breakage of chemical bonds within the DNA as well as by the generation of free radicals (107, 108). Among the diverse damaging effects of IR, the induction of DNA double strand breaks is considered as the most toxic lesion in cells.

The hematopoietic compartment is particularly sensitive to IR, for example, blood sample analysis revealed the rapid development of a hematopoietic syndrome in patients exposed to a total body irradiation of 1–2 Gray (Gy), which was characterized by a decline of the hematopoietic compartment (109). Similar to other hematopoietic cells, lymphocytes are particularly sensitive to radiation-induced cell death. Nevertheless, the various lymphocyte subtypes differ in their radiosensitivity: up to now it has been demonstrated that B cells, naive T cells, and NK cells are highly radiosensitive, whereas T memory cells, NK T cells, and T_reg_ cells are more resistant to the toxic effects of IR (110–114). Moreover, a study from 1995 revealed a higher radiosensitivity of IL-4-producing T_H_2 cells compared to T_H_1 cells (115). Based on their high radiosensitivity and easy accessibility, blood lymphocytes are frequently used in biodosimetry (116–119).

Further direct effects described in irradiated lymphocytes concentrate on the transcriptional response of these cells to IR, e.g., by gene expression profiling. These studies revealed that a majority of the strongly activated genes are p53 targets, like DNA damage-binding protein 2, the BCL-2-associated gene BAX, and tumor necrosis factor receptor superfamily, member 10b (TNFRSF10B) that are involved in DNA repair and apoptosis regulation (120–122).

However, in addition to these direct or “targeted” effects of IR on lymphocytes such as the induction of cellular stress responses and cell death in lymphocytes within the radiation field, lymphocytes can also mount an indirect response to radiation-induced tissue damage. In this context, “danger signals” released from damaged or dying cells in irradiated tissues result in lymphocyte activation, infiltration into the damaged tissue, and the release of inflammatory mediators (Figure 2).

Of note, high-dose irradiation also efficiently triggers direct tumor cell death and augments innate immune responses and tumor-specific immunity thereby enhancing the local and distant antitumor effects of RT. This was nicely summarized by one of the pioneers in the filed of radioimmunotherpy, Silvia Formenti (123). Accordingly, high numbers of tumor-infiltrating cytotoxic lymphocytes may predict the response of certain tumors to treatments involving RT (105, 124). This is a hot topic in the field of radiation biology and oncology highlighted in other reviews; here, we will concentrate on the contribution of radiation-induced modulation of the lymphocyte compartment to the adverse late effects of IR in the lung.

## Role of Lymphocytes in the Irradiated Lung

As nicely highlighted in a recent review on the general effects of IR on T lymphocytes and normal tissue responses, our understanding of the interaction between lymphocytes and radiation-induced tissue damage is still rudimentary (125). Because of the complexity of the involved cellular systems and soluble factors, investigations about the mechanisms underlying radiation-induced adverse late effects, for example, in the lung can only be performed in patients as well as animal models *in vivo*, particularly rodent models.

### Importance of the Experimental Model

Experimental models that use a single high-dose whole thorax or hemithorax irradiation of fibrosis-sensitive mice (C57BL/6) mimic human disease with respect to the time course and major symptoms of the disease (pneumonitis, fibrosis) and are therefore frequently used to study the underlying mechanisms, to define disease biomarkers and novel therapeutic targets, and to explore potential toxic effects of new combination strategies using IR in combination with molecularly targeted drugs (26, 126–130).

In this context, it is important to consider that mice with different backgrounds differ in their sensitivity to acute and chronic responses (pneumonitis, fibrosis), and these differences seem to be associated with differences in immune response in fibrosis-sensitive (C57BL/6, C57BL/6J) and fibrosis-resistant mice (e.g., BALB/c, C3Hf, and A/J) (131–133). In support of this assumption, a comparison of the transcriptome of lung tissue of fibrosis-prone C57BL/6J or fibrosis-resistant C3Hf/KAM mice upon BLM treatment revealed that the differences between the mouse strains included genes important for apoptosis, oxidative stress, and immune regulation (134).

On the other hand, due to the long latency of radiation-induced adverse side effects, researchers frequently use local or systemic administration of the DNA-damaging drug BLM as a radiomimetic drug that rapidly provokes a pronounced lung fibrosis in rodent models (135). However, work from our own group and from others suggests that the impact of the immune system on disease outcome may play different roles in the BLM and RT model, particularly when the acute model of intratracheal application of BLM is used (35, 136, 137). Since the C57BL/6 model is suited for the analysis of both, pneumonitis and fibrosis, we focus particularly on this model in the subsequent paragraphs. But, we are aware of the fact that future studies are needed in a more clinically relevant setting with fractionated irradiation.

Investigations in rodent models using experimental whole thorax or hemithorax irradiation are still underrepresented. Thus, preclinical studies about the contribution of RT-induced immunomodulation in normal tissues to radiation-induced lung disease are rare particularly with respect to lymphocyte responses. We therefore included data about lymphocytes responses from studies in other models of chronic respiratory disease or pulmonary fibrosis, where appropriate. We are aware that the described models are different with respect to the initial injury, the time course, and some of the involved mediators. However, from an immunological point of view, the different models of (chronic) inflammation/fibrosis share a sterile inflammation/repair/remodeling response to an initial damage/trauma and will therefore help to understand lymphocyte responses in the lung in response to radiation-induced tissue damage.

### Early Effects of Thoracic Irradiation on Lymphocytes in the Lung

Early immune suppression with subsequent lymphocyte infiltration are common responses of irradiated tissues during the acute and chronic phase after irradiation, including the lung tissue (40, 42, 138, 139). Preclinical studies in mice corroborated the infiltration and reconstitution of lymphocytes observed in patients (for more details, see Table 1). For example, Paun et al. analyzed the primary radiation injury response of the lung at 6 h, 1, and 7 days after 18 Gy whole thorax irradiation in different mouse strains and characterized infiltrating T cell populations and their cytokine profile in the lung tissue and in the bronchoalveolar lavage fluid (BALF). The authors reveal lower T cell levels in the BALF and increased numbers of infiltrating T_H_1 and T_H_2 cells in the lung at day 1 and 7 (140). Another murine study from Zheng et al. uncovered a more delayed reconstitution of CD4^+^ T cells compared to that of CD8^+^ T cells upon low dose total body irradiation (2.5 Gy); furthermore, T_H_1 reconstitution was also impaired, whereas T_H_17 and T_reg_ cells were elevated (141). In an own study, we showed that a 15 Gy whole thorax irradiation in mice led to a slight decrease in the percentage of CD3^+^ T cells in the lung at day 10 and 21 post-irradiation. In line with these findings, we found significant decreased levels of CD3^+^ T cells, including CD4^+^ and CD8^+^ T cells, at these time points in peripheral lymphoid organs like cervical lymph nodes and the spleen (142).

**Table 1:** **Lymphocytes in the irradiated lung**.

Background	Cell type in the lung [days (d) post-irradiation]	Disease stage	Reference
Murine model	T_H_1 (CD4^+^ IFN-γ^+^) ↑ (d1, d7)	Acute radiation response	(140)
Thorax XRT	T_H_2 (CD4^+^ IL-13^+^) ↑ (d1, d7)		
18 Gy	T_H_17 (CD4^+^ IL-17^+^) ↓ (d1, d7)		

Rat model	CD4^+^ ↑ (d28)	Pneumonitis	(43)
Thorax XRT			
Unilateral			
20 Gy			

Murine model	T_H_17 associated ↑	Pneumonitis	(46)
Thorax XRT	(IL-17, IL-23, IL-27) (d21)		
15 Gy	T_reg_ ↑ (d21)		

Murine model	T_reg_ ↑ (d21)	Pneumonitis	(142)
Thorax XRT	CD4^+^ ↑ (d42, d84)		
15 Gy			

Murine model	T_reg_ ↑ (d30, d90, d180)	Pneumonitis	(185)
Thorax XRT		Fibrosis	
20 Gy			

Murine model	CD3^+^ ↑ (BALF) (d56, d112, d168)	Pneumonitis	(38)
Thorax XRT		Fibrosis	
15 Gy			

Murine model	T_reg_ ↑ (d14, d30, d90, d180)	Pneumonitis	(81)
Thorax XRT		Fibrosis	
20 Gy			

Murine model	T_reg_ ↑ (d210)	Fibrosis	(35)
Thorax XRT			
15 Gy			

Patient study	CD4^+^ ↑ (BALF) (d30–d90)	Pneumonitis	(40)
2 Gy/day, 5 days/week, total 45–50 Gy			

Patient study	CD4^+^ ↑ (BALF) (d14)	Pneumonitis	(42)
2 Gy/day, 5 days/week, total 50–60 Gy	CD8^+^ ↑ (BALF) (d14)		

Patient study	CD4^+^ ↑ (BALF) (d15)	Pneumonitis	(39)
1.8–2 Gy/day, 5 days/week, total 45–50 Gy	CD8^+^ ↑ (BALF) (d15)		

*Murine (C57BL/6), rat, and patient studies revealing the presence of T lymphocytes during radiation-induced early and late adverse effects in the lung*.

Altogether, these studies highlight that lymphocyte subsets differ in their rates of radiosensitivity, recovery, and infiltration during different disease stages suggesting that they may have a distinct contribution to the dynamic changes in the environment in the irradiated lung tissue.

### Lymphocyte Responses to Signals from the Irradiated Lung

#### Damage-Associated Molecular Patterns (DAMPs)

In the past decade, several studies revealed how the immune system recognizes sterile tissue damage. Bianchi demonstrated how sterile tissue stress and damage in general led to the release of DAMPs (143). These endogenous “danger signals” induce and dictate an immune response to orchestrate repair, growth, and tissue homeostasis after damage (144, 145).

Besides this direct effect of IR, the response of the damaged resident tissue cells toward irradiation, e.g., epithelial cells, endothelial cells, or smooth muscle cells, involves a systemic “danger signal” that orchestrates immune cell recruitment and tissue repair (34). The radiation-induced oxidative injury in the lung and the release of DAMPs induce resident cells to secrete inflammatory and chemotactic cytokines. Human and murine studies revealed that DAMPs in the injured lung include among others extracellular heat shock proteins, S100 proteins, defensins, high-mobility group box-1 (146), extracellular nucleotides and nucleosides (35), as well as extracellular matrix (ECM) components like fibronectin, hyaluronan, uric acid, and surfactant proteins (37, 147). This is in line with findings from other studies dealing with lung injury, induced, for example, by smoke (148) or mechanical ventilation (149). Furthermore, animal studies with BLM-induced alveolitis revealed elevated levels of hyaluronan in the BALF and lung tissue on day 5 and was paralleled by an increased influx of polymorphonuclear leukocytes in the BALF and an interstitial–alveolar edema (150, 151).

For radiation-induced lung injury it has been described that the released DAMPs act through signaling *via* P2X, P2Y receptors, toll-like receptors (TLR-2 and TLR-4), receptor for advanced glycation end-products, and NOD-like receptors (NLRP3), respectively (152). Consistent with a role of TLR signaling in radiation-induced lung disease, *Myd88* knockout mice displayed increased as well decreased fibrosis development depending on the type of injury induced. In a BLM-induced lung injury model, *Myd88* knockout mice showed attenuated fibrosis and reduced cell infiltration in contrast to WT mice (136). In contrast, a study from Brickey et al. revealed that *Myd88* was protective in irradiated lungs and that irradiated *Myd88*^−/−^ mice had increased pro-fibrogenic factors and T_H_2 cytokines and displayed enhanced fibrosis levels at 24–27 weeks post-irradiation compared to WT mice (137).

Thus, the release of DAMPs, activators of an inflammatory cascade, leads to recruitment of inflammatory cells, leading to tissue inflammation, the so-called radiation-induced acute phase.

#### Cytokines/Chemokines with Impact on Lymphocyte Recruitment or Function

Moreover, thoracic irradiation triggers a rapid upregulation of the transcriptional regulator NFκB resulting in the production of pro-inflammatory cytokines (e.g., IL-6, IL-1α, IL-1β, TNF-α, and IFN-γ) within minutes to hours post-irradiation, at least at the mRNA level (34, 133, 153–155). However, as highlighted above, mouse strains differ in their cytokine profile after lung irradiation. Radiation induced distinct temporal changes in diverse cytokines in the pulmonary fibrosis-sensitive C57BL/6 mice compared to C3H mice (133). Rübe and colleagues demonstrated in a murine study that cells from the bronchiolar epithelium are a source for IL-6, TNF-α, and IL-1α in irradiated lungs (31). In line with these observations, Ao et al. described elevated IL-6 levels in the lungs of C57BL/6 model at 6 h post thorax irradiation with 12 Gy. In contrast, Paun et al. did not detect an increase in IL-6, IL-1β, IL-13, IL-17, and IFN-γ after 6 h post thorax irradiation with 18 Gy. These conflicting data highlight that the early stress response of the irradiated lung tissue requires further definition.

It is assumed that secreted mediators recruit immune cells, including neutrophils, granulocytes, macrophages, and lymphocytes, into the damaged tissue. In this context, a recent murine study described that the radiation-induced early lung inflammation was accelerated by induction of the inflammasome (Nlrp3, caspase 1, IL-1α, and IL-1β), highlighting the contribution of an early innate response (156). Exposure of lung tissue to IR triggered an increased influx of lymphocytes (30, 39, 140, 157). One of the driving forces of lymphocyte infiltration into the lung is the chemokine (C-C motif) ligand 18 (CCL18). Patients suffering from different lung diseases displayed elevated levels of CCL18, and these were associated with T cell recruitment (158–160). CCL18 is also known as *pulmonary and activation-regulated chemokine*; overexpression of CCL18 by intratracheal instillation of adenoviral vector AdV–CCL18 and subsequent overexpression of CCL18 in a murine BLM-induced injury model uncovered that CCL18 is highly selective for T cells and attracts these cells into the injured lung (161). Other potent chemoattractants that have been described to induce lymphocyte recruitment to a damaged lung tissue include the monocyte chemoattractant protein 1, IL-16, thymus and activation-regulated chemokine, macrophage-derived chemokine, CCL1 (I-309), CCL5 (RANTES), and stromal cell-derived factor 1 (46, 154, 162–164). In the injured tissue, the infiltrating lymphocytes get activated, start secreting diverse mediators, and finally contribute to a complex inflammatory milieu characteristic for pneumonitis.

As described above, various factors in the changing microenvironment of the irradiated lung have an impact on the response of recruited T lymphocytes that can act either in a pro- or in an anti-inflammatory way. For example, Paun et al. investigated pulmonary T helper cell populations during the acute radiation response of the lung after 18 Gy whole thorax irradiation in C57BL/6 mice and uncovered that T_H_1 (CD4^+^ IFN-γ^+^) and T_H_2 (CD4^+^ IL-13^+^) cells were increased at 1 and 7 days, but not at 6 h post-irradiation. Furthermore, they found a decrease in T_H_17 (CD4^+^ IL-17^+^) cells at 6 h, 1, and 7 day post-irradiation (140). Own investigations revealed the appearance of IL-17-expressing CD4^+^ T cells and CD4^+^FoxP3^+^ T-lymphocytes in the lung tissue of C57BL/6 mice at 21 days after whole thorax irradiation with a single high dose of 15 Gy (46).

During the early pneumonitic phase between 3 and 12 weeks post-irradiation (127) recruited T lymphocytes secrete T_H_1-like, pro-inflammatory TNF-α, IFN-γ, IL-2, and lymphotactin to attract and activate more immune cells to the site of damage (165). IFN-γ, for example, activates “classically activated” macrophages (M1) with high nitric-oxide synthase 2 expression but, on the other hand, shows suppressive effects on myofibroblasts and inhibits the production of ECM proteins (166, 167). Besides this classical T_H_1 response, a pro-inflammatory T_H_17-dominant response was observed after thoracic irradiation in mice. Cytokine levels of IL-16, IL-17, IL-23, and IL-27 were elevated 3 weeks post-irradiation where they might promote chronic inflammation and tissue damage (46).

The observed findings reveal that besides the early induction of cytokines like IL-1, IL-6, and TNF-α primarily lymphoid T_H_1 and T_H_17 responses contribute to the pro-inflammatory, pneumonitic phase.

### Reconstitution of Lymphocytes in the Irradiated Lung

Of course, the imbalance in the hematopoietic compartment after exposure to IR needs to be restored. Interestingly, the overall recovery rate of different lymphocyte subsets varies in different organs and is chemokine dependent (168). For example, Santin et al. analyzed blood samples from irradiated patients with squamous cervical cancer: in this study CD8^+^ cells recovered in the blood faster than CD4^+^ cells over a time period of 40 days after a 5-week radiation treatment (169). This is consistent with earlier studies suggesting a prolonged reduction in lymphocyte proliferation, a persistent reduction in cell counts, or even an inversion of the CD4^+^/CD8^+^ ratio after whole body irradiation and thorax irradiation in breast cancer patients (170–173). Furthermore, in a recent study with 1,423 lung cancer patients, Yan et al. found significantly increased levels in blood CD3^+^ T-cells, especially CD8^+^ T cells, compared to CD4^+^ T cells in the patients 3 months after RT of the lung (174). Analysis of total lymphocytes in limited-stage small cell lung cancer (LS-SCLC) patients revealed a decrease in total lymphocytes during RT followed by recovery after the end of treatment; interestingly, the authors correlated the severity of radiation-related lymphopenia to treatment outcome revealing a potential use of radiation-related lymphopenia for prediction of poor survival in LS-SCLC (175).

### Chronic Effects of Thoracic Irradiation on Lymphocytes in the Lung

#### T_H_ Subsets

Substantial changes in the lung environment are observed during the chronic inflammatory and fibrotic phase in the irradiated lung. It is thought that in this context the shift from a T_H_1 cytokine profile toward a T_H_2 cytokine profile could be a key event. The signature of T_H_2 cytokines IL-4, IL-5, IL-10, and IL-13 is known to convey strong anti-inflammatory and pro-fibrotic effects, to mediate fibroblast activation, and activate “alternatively activated” macrophages (M2) with high arginase-1 expression (43, 166, 176, 177). Of further interest is a recent study from 2016 showing that radiation-induced lung fibrosis in a tumor-bearing mouse model was associated with enhanced type 2 immunity (178). In this study, the authors revealed that GATA3 expression in tumors appeared to affect the response to the normal lung tissue to radiation-induced damage. Lung fibrosis was more severe in tumor-bearing mice than in normal mice post-irradiation, highlighting that the enhanced type 2 immunity in tumors appeared to influence the outcome of radiation damage.

Despite the known pro-fibrotic actions of the IL-4/IL-13 axis, a recent study suggested a potential protective role for the IL-13Rα1 in a murine model of BLM-induced fibrosis; here, the authors speculated that IL-4 and/or IL-13 may act through the type 2 IL-4 receptor to regulate epithelial cell healing and immune responses to lung damage and further protection against pulmonary fibrosis (179). In line with this study, Han et al. also identified increased expression of the type 2 key transcription factor GATA3 in C57BL/6 mice that had received 12 Gy thorax irradiation (176).

Interestingly, first data about the role of the subset of T_H_9 cells have been obtained in a model of pneumonitis and fibrosis induced by silica particles; these suggest that IL-9 expression may reduce lung fibrosis and type 2 immune polarization (180). Taken together, until now the impact of the cytokine production of T_H_1, T_H_2, T_H_9, and T_H_17 subsets in radiation-induced pneumopathy has not been fully described and understood, but it is highly likely that T_H_2-driven responses are a key event of radiation-induced fibrosis development.

Up to now, there is only limited information available about the impact of the B cell compartment on the outcome of radiation-induced pneumopathy and other models of fibrotic lung disease. A recent transcriptome analysis of irradiated mouse lungs at 24 weeks after exposure to IR revealed that genes associated with B cell proliferation and activation were significantly induced in irradiated lungs of fibrosis-prone C57BL/6 mice suggesting a possible role of B cells in radiation-induced pneumopathy (181). Interestingly, deficiency in the B cell surface molecule CD19 in mice reduced the susceptibility to BLM-induced fibrosis, whereas CD19 overexpression in mice aggravated BLM-induced fibrosis (182). In contrast, B cells under the influence of IL-9 participated in the protection against lung fibrosis in a murine model of silica particle-induced lung fibrosis, and their protective effect was associated with the overexpression of prostaglandin-E2 (PGE2) in macrophages (183). Interestingly, PGE2 itself is thought to be anti-fibrotic due to its suppressing effects on fibroblast proliferation and its ability to reduce the expression of collagen mRNA (184). These controversial findings highlight the need for further studies to clarify the protective or destructive role of B cells in radiation-induced pneumopathy and the involved mediators.

#### TGF-β and T_reg_

Besides the influx of T helper lymphocytes, the T_reg_ subset also infiltrates the irradiated lung tissue, where it is thought to suppress an exaggerated inflammation (80, 81, 142, 185). T_reg_ can be generally induced by TGF-β (186). This cytokine is released early after tissue injury by type II pneumocytes, fibroblasts, and immune cells (187, 188), but the latent form can also be activated by radiation *in vitro* (189). During this early phase TGF-β is thought to function mainly as a pro-inflammatory mediator to attract neutrophils but may also provide signals for limitation of tissue inflammation, e.g., by inducing T_reg_ (190, 191). During the later remodeling phase (24–30 weeks), macrophages can be a source for TGF-β; during this phase TGF-β is known to promote repair processes and to favor fibrosis. This might explain the observed biphasic appearance of T_reg_ in the acute and in the chronic phase of radiation-induced lung injury described recently (35, 142, 185). *Vice versa*, T_reg_ can also produce TGF-β as well as IL-10, revealing their suppressive capacity and suggesting an additional pro-fibrotic action (79, 192). In this context, it is discussed that besides TGF-β epithelial cell-derived IL-18 and IL-33—released after tissue damage—might be also important for the induction and maintenance of T_reg_. The activation of T_reg_ *via* IL-33 and its receptor *suppression of tumorigenicity* 2 (ST2, also known as IL33R, IL-1RL1) lead to a T_H_2-like character, expressing GATA3 and secreting T_H_2 cytokines IL-5 and IL-13. Furthermore, IL-18- and IL-33-activated T_reg_ showed higher suppressive capacity by enhanced activation and secretion of the anti-inflammatory and pro-fibrotic cytokines IL-10 and TGF-β as well as amphiregulin (AREG) (193–195). Thus, we speculate that IL-18/IL-33-driven T_reg_-activation contributes to tissue repair and a pro-fibrotic actions in the lung. Further studies are needed to confirm this in a radiation-induced lung injury model.

It has been shown that T_reg_ contribute to fibrotic diseases in the lung such as radiation-induced, BLM-induced lung injury and IPF by modulating the microenvironment through mechanisms involving among others induction of Th17 responses, shifting the IFN-gamma, IL-12/IL-4, IL-5 balance, and promoting endothelial to mesenchymal transition (79–81, 196, 197).

Nevertheless, the role of T_reg_ in pneumopathy remains to be further elucidated as these cells play distinct roles in different disease stages and disease models (79, 80, 192, 196, 198, 199). While depletion of CD4^+^CD25^+^ T cells with an anti-CD25 antibody during an early stage of BLM-induced lung disease reduced the levels of inflammatory cells, collagen deposition, TGF-β, and lung fibrosis in mice, T_reg_ depletion during later stages in this model led to a more pronounced infiltration of inflammatory cells and increased fibrosis scores (196). This highlights a disease-promoting effect of CD4^+^CD25^+^ T cells during the acute phase of BLM-induced lung disease and a protective role of these cells during the fibrotic phase. By contrast, abrogation of the long-lasting (6 months) increase in CD4^+^CD25^+^ T_reg_ observed in irradiated lungs of C57BL/6 mice by long-term CD4^+^CD25^+^ T cell depletion with an anti-CD25 antibody reduced the increase in fibrocytes and attenuated radiation-induced lung fibrosis (80). In this model, depletion of CD4^+^CD25^+^ T cells covered both, the pneumonitic and the fibrotic phase so that the two publications are not directly comparable. Nevertheless, the latter report implicates that a disease-promoting effect of CD4^+^CD25^+^ T cells is predominant in radiation-induced lung disease. To our present view, the suppressive properties of T_reg_ are needed during the pneumonitic phase to dampen an overwhelming and excessive pro-inflammatory response that is however initially needed to induce repair and regeneration. Yet, during the chronic disease stage under the influence of a changing environment, T_reg_ seem to adopt a pathologic character, secreting mediators like IL-10, TGF-β, and AREG, thereby contributing to a fibrosis-promoting intercellular cross talk. Our current hypothesis of the underling mechanisms is summarized in Figure 2; depending on the cell type the persistent damage caused by irradiation of resident cells will result either in a delayed partial cell loss (e.g., endothelial cells, alveolar epithelial cells) or in a chronic cell activation (e.g., MSC, fibroblasts) thereby driving chronic environmental changes (e.g., chronic increase in tissue hypoxia, adenosine, hyaluronan, macrophage-derived IL-10 and TGF-β, and epithelial-derived IL-18/IL-33) that promote among others the generation and a phenotypic adaptation of T_reg_. Under such conditions, the phenotype of ILC—like that of T_reg_ cells and myeloid cells—may shift toward a disease-promoting phenotype supporting chronic lung inflammation and pulmonary fibrosis in irradiated lung tissue. It is therefore highly likely that the distinct roles of lymphocytes and T_reg_ in BLM-induced versus radiation-induced pulmonary fibrosis may be due to differences in impact of an acute but reversible damage by the drug BLM and a chronic, persistent impact of IR on the environmental changes and associated immune changes.

The findings reported so far highlight the need for more detailed mechanistic analyses about the role of T_reg_ for adverse late effects of IR in the lung.

## Therapeutic Approaches for Radiation-Induced Pneumopathy

There is increasing evidence that lymphocytes play a role in RT-induced adverse late effects in the lung. Thus, these cells or the mediators associated with their pro- or anti-fibrotic function may constitute valuable targets for the prevention or treatment of radiation-induced lung disease. However, so far there is only little knowledge about the use of specific lymphocyte subpopulations or their mediators as potential diagnostic or predictive biomarkers for early or late adverse effects of IR in the lung. Consequently, so far treatment strategies targeting immune cells or associated mediators suspected to participate in disease pathogenesis are only tested in preclinical investigations in mice.

Instead, current treatment options for patients suffering from radiation-induced pneumonitis are limited to the symptomatic administration of anti-inflammatory drugs such as glucocorticoids thought to limit the toxic effects of the overwhelming inflammation by reducing the levels of pro-inflammatory cytokines, chemokines, and growth factors. But, these anti-inflammatory therapies may also indirectly impact on lymphocyte responses, their activation state, or both.

### Current Treatment with an Impact on Lymphocyte Responses

Experimental studies in patients mostly aim or address either the impact of the treatment on radiation-induced pneumonitis or on radiation-induced lung fibrosis, respectively. However, immunomodulatory strategies will mostly influence both, early and late disease stages. For example, treatment of patients developing pneumonitis with anti-inflammatory drugs such as glucocorticoids and pentoxifylline (PTX) will also impact on the chronic radiation-induced immune changes thereby potentially influencing progression to lung fibrosis.

Due to their anti-inflammatory and immunosuppressive properties, glucocorticoids such as dexamethasone and prednisone are widely used after lung irradiation in symptomatic patients. Both drugs affect the expression of inflammation-associated genes by interaction with the steroid receptor. For example, glucocorticoid treatment involves the inhibition of the NF-κB pathway (200) as well as inhibition of the expression of IL-17A, TGF-β, IL-6, and TNF-α, thereby reducing radiation-related inflammation (201). Furthermore, dexamethasone also reduced the deposition of collagen in the lung tissue of irradiated mice (201, 202).

Pentoxifylline and alpha-tocopherol (vitamin E; Vit E) also exert anti-inflammatory actions and have already been used in patients. PTX is a xanthine derivative that acts by inhibiting TNF-α, IL-1, fibroblast growth factor, TGF-β as well as the SMAD pathway, whereas Vit E is known to counteract TGF-β and the SMAD signaling (203). In a study with 40 patients PTX was shown to provide significant protection against the early and late adverse late effects of RT in the lung (204). Furthermore, in another randomized trial study radiation-induced adverse effects were more frequent for all disease stages in the untreated control group of patients receiving irradiation alone compared to the groups where irradiation was combined with PTX and Vit E (205).

Another interesting approach that is already being explored since 1990 is the use of *angiotensin-converting enzyme* (ACE) inhibitors to treat radiation-induced adverse late effects in the lung (206). Interestingly, these inhibitors also interfere with immune responses, e.g., in T lymphocytes (207, 208). The membrane-bound ACE hydrolyzes a spectrum of substrates with physiologic relevance like angiotensin, bradykinin, or neurotensin (209) and is expressed in human CD4^+^ and CD8^+^ T-lymphocytes but not in B cells (210). Investigations with ACE inhibitors like captopril and ramipril in patients revealed a potential benefit in decreasing the incidence of radiation-induced pneumonitis (211–214). Unfortunately, a recent clinical study from 2016 validating the protective effect of the ACE inhibitor captopril in radiation-induced lung toxicity failed due to low accrual and a high number of patients who had to be excluded from the analysis. Nevertheless, the study confirmed safety of the ACE inhibitor treatment; the authors suggest that the use of newer ACE inhibitors (e.g., enalapril or lisinopril) during RT may be suited to solve the problems identified in their trial (215).

Other immunosuppressive agents that have been tested earlier preferentially in single case reports are cyclosporin and azathioprine. Cyclosporin is a common immunosuppressive agent that acts on CD4^+^ T lymphocytes by inhibiting the transcription of the interleukin-2 gene (216). Cyclosporin has already been described in 1991 as a treatment option in interstitial lung disease (217). Moreover, treatment with cyclosporine successfully reduced the symptoms of radiation-induced pneumonitis in a case study (218). Instead, case studies testing the use of azathioprine, a drug with suspected inhibitory effects on T lymphocyte formation and B lymphocyte proliferation (219, 220), revealed either a beneficial effect (221) or no effect in radiation-induced pneumonitis (222).

So far, current treatment options are limited and focus on the control of overwhelming pro-inflammatory responses during the pneumonitic phase. Furthermore, risk factors are not well understood, and predictive biomarkers are lacking. This highlights the urgent need for further preclinical and clinical studies to gain a more comprehensive understanding of the underlying mechanisms as well as the complex cellular cross talk and the mediators driving disease pathogenesis, if we aim to define predictive biomarkers and novel therapeutic approaches.

### Current Research and Future Perspectives

Anti-inflammatory drugs are effectively reducing the symptoms of radiation-induced pneumonitis, and most patients completely recover from pneumonitis. However, many patients suffering from thorax-associated neoplasms, particularly lung cancer, or patients with chronic respiratory disease have an increased risk to develop lethal pneumonitis. Therefore, it is important to uncover diagnostic and prognostic biomarkers for radiation-induced lung disease, particularly lethal pneumonitis. Several patient-associated factors have been described to be associated with an increased risk to develop (severe) radiation pneumonitis such as the patient age and fitness, additional disease (e.g., tumor, COPD), as well as treatment (e.g., XRT fractions, drug administration) (223–225). Furthermore, several potential biomarkers such as pulmonary function, dose–volume histogram, end/pre-RT plasma levels of TGF-β1, IL1α, and IL6 have been described that may be suited to predict a higher risk for radiation-induced lung toxicity (226, 227). A promising novel approach to predict and understand genetic risk factors for radiation-induced toxicity may be the use of radiogenomics (228).

Despite the high efficacy of anti-inflammatory drugs in reducing the symptoms of radiation-induced pneumonitis, patients recovering from pneumonitis still have the risk of developing subsequent pulmonary complications. However, effective mechanism-based options for the treatment of pulmonary fibrosis are still limited (229). Therefore, another important topic of current research is to develop effective therapeutic strategies to prevent or treat radiation-induced lung fibrosis (223, 230); experimental approaches mostly target radiation-induced formation of free radicals, cell death, or specific cytokines or growth factors, respectively.

Importantly, a review from 2010 highlighting patient data as well as experimental studies mentioned already that “*One last target that may need further investigation is that of the immune system, more specifically the alteration in immune responses*…” (231). As shown in Table 1, the majority of studies in public databases identified CD4^+^ T cells and T_reg_ as key populations among lymphocytes infiltrating lungs during radiation-induced pneumonitis and fibrosis. Though molecular approaches to reduce radiation-induced lung fibrosis by inhibiting pro-fibrotic mediators (e.g., TGF-β) are known to modulate lymphocyte induction/activation (232), 6 years later, our knowledge about the role of diverse lymphocytes during fibrosis is still limited. Targeted approaches to modulate lymphocyte recruitment, activation, or signaling are rare and still limited to preclinical studies in various murine models of chronic respiratory disease or fibrosis-associated disease, respectively.

One promising approach is to target the cytokine IL-17A as it has been suggested that the protective effects of anti-inflammatory drugs such as dexamethasone involve the reduction of IL-17A (233). In this context, treatment with an antibody against IL-17A reduced IL-17A, TGF-β, and IL-6 concentrations and alleviated radiation-induced pneumonitis and subsequent fibrosis in mice (233). Another interesting approach to target IL-17 might be the use of phosphodiesterase-4 inhibitors such as roflumilast that prevent the breakdown of cAMP thereby inhibiting fibroblast activation and TGF-β induction (234). In a murine model of chronic asthma, treatment with roflumilast reduced the expression of IL-17A, TNF-α, GM-CSF, and IL-6 to a similar extent as dexamethasone implying a potential use of roflumilast for the treatment of adverse events in the irradiated lung (235).

Another potential target currently in the focus of research of other diseases including cancer are T_reg_. As mentioned above, T_reg_ can be induced by TGF-β and also secrete or bind TGF-β (236, 237). This suggests that targeting T_reg_ might be suited to counteract radiation-induced adverse late effects in the lung and other diseases rich in tissue TGF-β-levels such as, skin, liver, and kidney (238–240). In support of this assumption, abrogation of the long-lasting (6 months) increase in T_reg_ by depletion with an anti-CD25 antibody counteracted the development of radiation-induced fibrosis in mice (80) thereby corroborating findings in other fibrotic diseases (79). Still, further studies are needed to strengthen these results with respect to radiation-induced (pulmonary) fibrosis. The above findings of Xiong et al. are of particular interest because several studies highlight the potential importance of T_reg_ depletion in enhancing antitumor immunity during RT (241, 242). One major challenge in targeting T_reg_ will be defining the optimal treatment schedule, since T_reg_ might also be beneficial in counteracting exaggerated inflammation during the pneumonitic phase (142).

In this context, we recently showed that the CD73/adenosine axis is a potential target in radiation-induced lung fibrosis. Lymphocytes, especially T_reg_, showed high CD73 expression after irradiation, and a CD73 deficiency in mice led to reduced expression of pro-fibrotic mediators like TGF-β and osteopontin during the fibrotic phase (35). Further unpublished data reveal that genetic deficiency of CD73 also precludes the accumulation of alternatively activated macrophages in prefibrotic macrophage clusters in the irradiated lung tissue (deLeve and Wirsdörfer, unpublished observations). The contribution of the CD73/adenosine pathway in fibrosis has already been described for BLM-induced pneumopathy and in other fibrotic diseases (243–245). Adenosine can bind to four different adenosine receptors to induce anti-inflammatory and pro-fibrotic actions. It is known that it inhibits lymphocyte proliferation, activation, and cytokine secretion. Furthermore, it promotes the induction and activation of T_reg_, highlighting its role in immunomodulation (246). In our hands, therapeutic targeting of the CD73/adenosine pathway by either enzymatic inhibition of adenosine accumulation or antibody blockade of adenosine-converting CD73 attenuated fibrosis development upon a single high-dose (15 Gy) whole thorax irradiation of C57BL/6 mice (35). The complex mechanism of the adenosine action in the pathogenesis of fibrotic diseases is not fully understood and is under current investigation. Importantly, the CD73/adenosine pathway has recently emerged as a novel immune checkpoint that tumor cells use to dampen intratumoral immune responses (247). Therefore, pharmacologic strategies for modulating CD73 or adenosine may limit radiation-induced adverse late effects presumably without increasing or even decreasing radiation resistance of tumor cells (35).

A novel potential candidate for future treatment options might be the group of ILC. ILC seem to play a critical role in lung inflammation, tissue remodeling, and fibrosis development thereby revealing a therapeutic potential of modulating ILC responses in the lung. Although it is highly likely that ILC may also impact radiation-induced lung disease, their role has not yet been investigated. Further studies are needed to clarify their contribution to acute and chronic disease stages after irradiation and to uncover a therapeutic potential in the context of ILC signaling, e.g., by targeting IL-33 or ST2. The cytokine IL-33 is important in innate and adaptive immunity and contributes to tissue homeostasis and is induced under environmental stress. IL-33 can be released by epithelial cells after tissue damage and is a trigger of tissue repair induced by ILC2 and T_reg_ (193). Regarding lung fibrosis, it was revealed that IL-33 enhanced BLM-induced fibrosis by increasing the levels of the T_H_2 cytokines IL-4, IL-5, or IL-13 (103), leading to the assumption that ILC2 or T_H_2 cells are induced. Moreover, it was shown that treatment with an anti-IL-33 antibody attenuated BLM-induced lung inflammation and fibrosis (104). A current review nicely summarizes the role of IL-33 and ST2 in health and disease highlighting its potential for therapeutic intervention also in fibrotic lung disease (193). The observations on IL-33 activity in the lung with an impact on T_H_2 responses and tissue repair make it highly likely that IL-33/ST2 signaling may also impact on radiation-induced lung fibrosis. Further studies are needed to clarify the role of this signaling axis in radiation-induced pneumopathy.

Another interesting and novel therapeutic option to treat radiation-induced adverse effects in the lung with a potential interaction with lymphocytes is the therapeutic application of MSC or of microvesicles/exosomes secreted by MSC (36, 248–252). This approach is based on the initial observation that healthy resident MSC are important to lung homeostasis and protect the lungs after injury among others by immunomodulation mostly through paracrine mechanisms (253–256). However, when resident-specific endogenous MSC are damaged or lost, e.g., by differentiation into myofibroblasts, this cell population contributes to TGF-β production, tissue remodeling, and fibrosis in various models, including thoracic irradiation, exposure to BLM, and IPF (37, 257–262). In this context, impaired regulation of effector T cell proliferation upon loss of resident pulmonary MSC has been implicated in the development of BLM-induced fibrosis (257).

Instead exogenously applied MSC may exert tissue protective effects by differentiating into an epithelium-like phenotype and replacing damaged cells, although our own data hint to a minor contribution of this effect to their protective effects (36, 263). Interestingly, recent findings highlight the ability of MSC to transfer (healthy) organelles or molecules by direct cell-to-cell contact through tunneling nanotubes or by the release of exosomes or microvesicles, respectively (255).

Several reports including own studies revealed that MSC show anti-fibrotic and protective effects in the irradiated lung, and current reviews highlight a potential therapeutic benefit of MSC therapy for the treatment of radiation-induced and BLM-induced tissue damage (36, 37, 248, 263). In our hands, adoptively transferred MSC normalized certain aspects of radiation-induced immune deviation in the lung tissue, normalized vascular function, and attenuated radiation-induced pulmonary fibrosis (36, 37). We and others showed that the anti-inflammatory and anti-fibrotic action of MSC is mediated by the inhibition of TNF-a, IL-1alpha, and interleukin 1 receptor antagonist (249, 251), stimulating the secretion of hepatocyte growth factor (HGF) and PGE2 (250, 252) and restoration of the superoxide dismutase 1 expression (37).

Generally, MSC might also exert protective effects during pneumonitis and fibrosis development due to their antiproliferative effects (253) and their suppressive capacity on innate and adaptive immune responses (254). The suppressive capacity on lymphocytes is mediated by the secretion of soluble factors like HGF, PGE2, truncated CCL-2, IL-10, and PD-1 ligation thereby inhibiting CD4^+^ T cell proliferation and the polarization toward a T_H_1 and T_H_17 phenotype (254). Due to a potential inhibition of T_H_1 and T_H_17 cells during radiation-induced pneumonitis, MSC might dampen an excessive immune response. This effect may be complemented by the ability of MSC to favor the development of a T_H_2 phenotype and T_reg_ (254, 264). This effect could be beneficial during the pneumonitic phase but may be disadvantageous during the fibrotic phase. We speculate that by dampening tissue inflammation and remodeling and restoration of resident cell function, MSC limit resident cell loss/dysfunction, chronic inflammation, and fibrosis-promoting environmental changes. In this context, others and we described that MSC treatment has protective effects in the lung due to a transdifferentiation into an epithelium-like phenotype (263) and by the protection from endothelial cell loss (37), respectively.

Finally, another experimental approach to reduce DNA damage-induced late effects in the lung with an impact on lymphocytes is the use of the lysophosphatidic acid receptor (LPA_1_) antagonist AM966: treatment with AM966 revealed reduced lung injury, vascular leakage, lymphocyte recruitment, and fibrosis development at 14 days after BLM treatment (265). However, this drug has not yet been tested in radiation-induced lung disease.

Nevertheless, current studies investigating the role of lymphocytes and their inducers and mediators as therapeutic targets in radiation-induced adverse effects in the lung are still rare. This might be due to insufficient knowledge on the beneficial and adverse role of specific lymphocytes subsets in different stages of disease pathogenesis.

## Final Remarks

Up to now, mechanistic knowledge about the role of lymphocytes in radiation-induced pneumopathy is still limited, and no reliable diagnostic or predictive biomarkers for beneficial and adverse effects of specific lymphocyte subsets are available to date. Nevertheless, preclinical and clinical investigations indicate that radiation-induced immune changes are important to the outcome of RT and that lymphocytes contribute to the beneficial and adverse effects of IR in tumors and normal tissues, including the lung.

We hypothesize that radiation-induced acute damage to resident cells including progenitor cells of mesenchymal origin and a perpetual cascade of cytokines/chemokines triggers immune cell recruitment and activation to promote tissue repair. However, in cases where the immune response cannot be controlled by anti-inflammatory cells such as T_reg_ or M2-like macrophages, patients may develop pneumonitis. If this initial damage response is not sufficient to repair the radiation-induced damage, the persistent damage results in chronic inflammation and delayed changes of resident cells, such as epithelial-mesenchymal-transition, endothelial-mesenchymal-transition, activation of MSC, or even chronic (mitotic) cell death of endothelial cells and alveolar epithelial cells. The resulting delayed environmental changes involve among others tissue hypoxia (by chronic endothelial cell loss), chronic inflammation, and chronic increase in fibroblasts and fibrosis-promoting mediators such as adenosine, hyaluronic acid, and TGFβ. These changes act together in the generation/activation of disease-promoting cell phenotypes such as activated myofibroblasts, pathologic T_reg_, or M2-like macrophages thereby promoting exaggerated ECM deposition and fibrosis development (Figure 2).

Here, we want to stress that radiation-induced immune changes can be either pro-inflammatory (acute phase) or anti-inflammatory/pro-fibrotic (chronic phase), and that lymphocytes exert distinct functions during radiation-induced pneumopathy (see Figure 1). We speculate that T_reg_ might be beneficial during radiation-induced pneumonitis due to their suppressive action on pro-inflammatory cells as seen in hypersensitivity pneumonitis (266). In contrast, as outlined above current preclinical studies in diverse murine fibrosis models highlight a potential contribution of T_reg_ in fibrosis development, and own work supports such a disease-promoting role also for radiation-induced pulmonary fibrosis (35). We therefore assume that these immunosuppressive cells or the environmental factors promoting their recruitment/expansion may constitute promising therapeutic targets to prevent or treat radiation-induced fibrosis. We are aware that the use of specific lymphocyte populations or associated signaling molecules as therapeutic targets is complicated by the fact these cells exert either beneficial or harmful roles. This depends on the disease stage, and potentially patient-specific genetic factors. Consequently this requires the careful definition of optimal treatment schedules to avoid immune cell-associated complications during both, pneumonitis and fibrosis. We also want to point out that recently discovered lymphocyte subsets like ILC as well as signaling pathways like IL-33/ST2 are of interest due to their potential contribution to RT-induced pneumopathy highlighting the need of related studies.

Further important issues that also need to be addressed are (i) the potential high intrinsic radioresistance of thorax-associated solid tumors treated by thoracic RT and (ii) a potential tumor immune escape. Therefore, it is important to consider that any inflammation-modulating or immune cell-targeting strategy for the treatment of radiation-induced pneumopathy may alter the antitumor effect of RT or combined treatment strategies involving immunomodulation or immunoboost; unfortunately, this issue is mostly not addressed by preclinical studies investigating the mechanisms of radiation-induced normal tissue toxicity. *Vice versa*, a potential increased normal tissue toxicity of such antitumor treatments is not analyzed when studying new combination treatments in preclinical models. Therefore, there is a high need to develop appropriate preclinical models if we want to identify treatment strategies balancing radiation-induced tumor cell clearance and normal tissue protection. Nevertheless, we are convinced that a better understanding of radiation-induced immunomodulation in tumors and normal tissues will offer novel opportunities for widening the therapeutic window by targeting immune cells or immune-associated mediators that promote both, tumor growth/resistance and normal tissue toxicity—of these, TGF-β and adenosine constitute perfect examples.

Taken together, it is important to deepen our knowledge about radiation-induced immune changes, including the modulation of lymphocyte recruitment, proliferation, and/or function of specific lymphocyte subsets during the different stages of radiation-induced lung disease. Further studies are needed to optimize therapeutic strategies for the prevention or treatment of adverse late effects of IR to normal tissues that also take into account the immune repertoire of the respective malignant disease before and after RT, if we aim at protecting the normal tissue without promoting tumor growth and *vice versa*.

## Author Contributions

FW and VJ equally contributed to the writing of this review article.

## Conflict of Interest Statement

The authors declare that the research was conducted in the absence of any commercial or financial relationships that could be construed as a potential conflict of interest.
